# Supplementary material for: USP8 Down-Regulation Promotes Parkin-Independent Mitophagy in the Drosophila Brain and in Human Neurons
Source: Cells. 2023 Apr 13;12(8):1143. doi: 10.3390/cells12081143 (PMC10136645; doi:10.3390/cells12081143)
Supplement: Supplementary file 1 [file cells-12-01143-s001.zip › cells-2201727 SM figures-main.pdf]

A

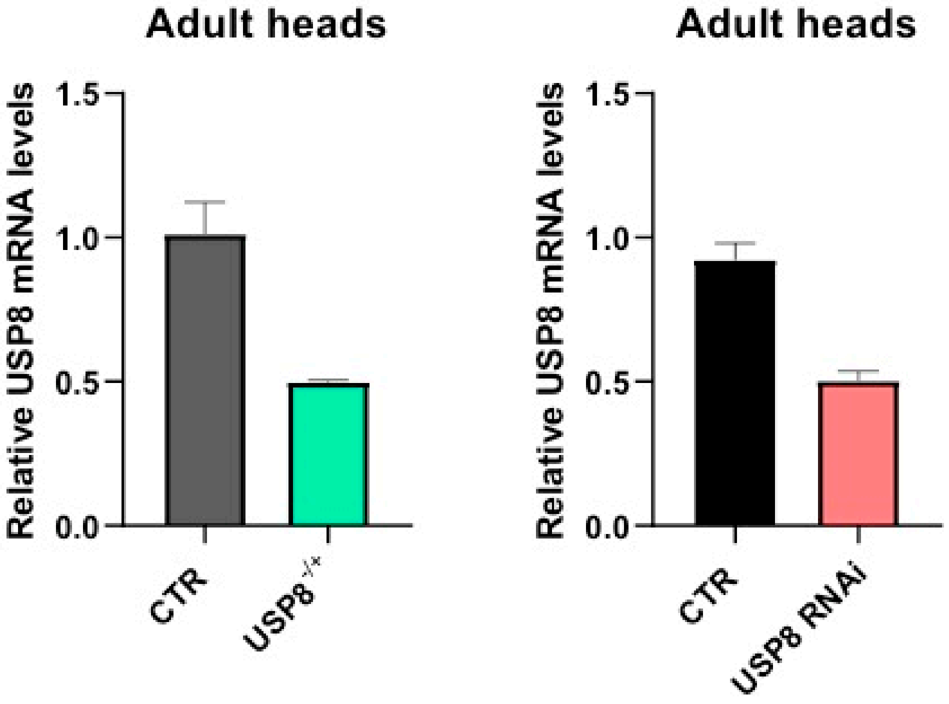

B

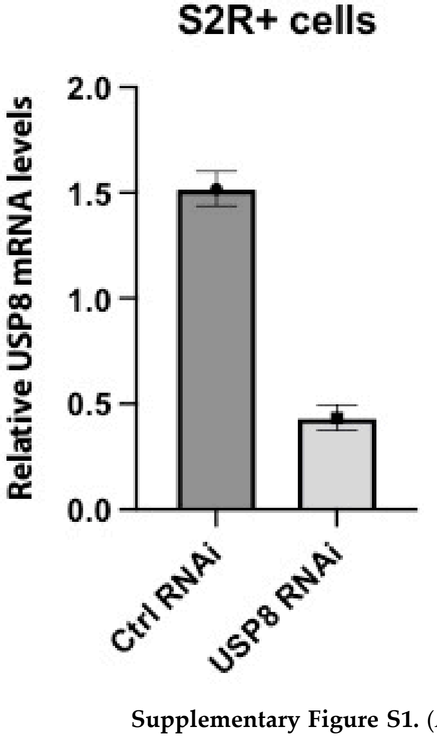

Supplementary Figure S1. (A) Total RNA was extracted from the heads of 3 days old flies of the indicated genotypes, and retrotranscribed into cDNA. Specific USP8 and endogenous control oligonucleotides primers were used to perform quantitative RT-PCR. Bar graph indicates USP8 mRNA levels relatively to endogenous control in USP8-/+ flies and nSybGAL4> UASUSP8 RNAi. (B) Total RNA was extracted from 3 days dsRNA treated S2R+ cells, and retrotranscribed into cDNA. Specific USP8 and endogenous control oligonucleotides primers were used to perform quantitative RT-PCR. Bar graph indicates USP8 mRNA levels relatively to endogenous control in dsRNA treated cells. Student's t-test, $P<0.0001$, $n=3$.

A

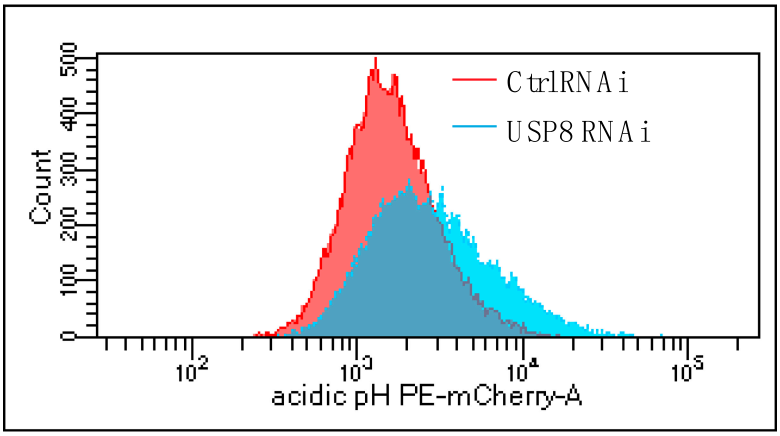

B

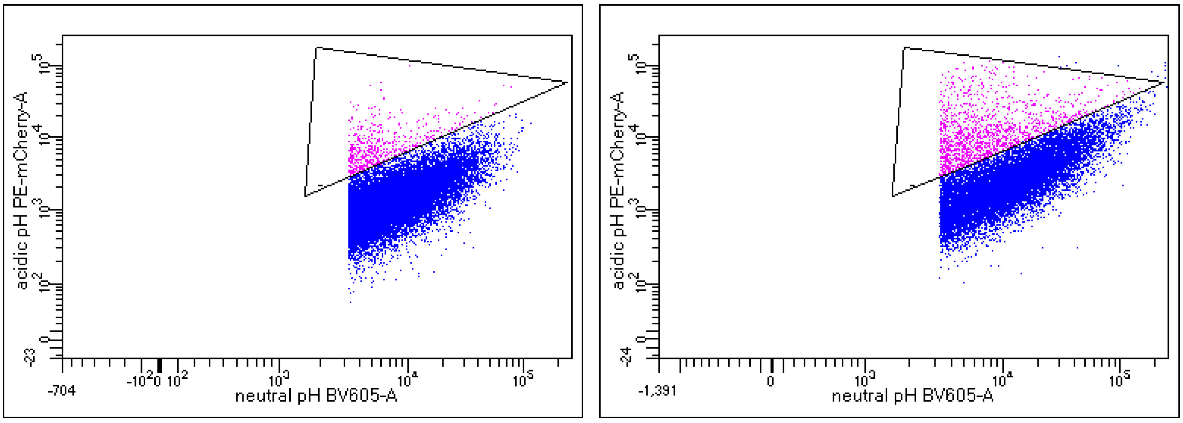

Supplementary Figure S2. Cells were treated with the indicated dsRNA, and after 24hrs they were transfected with mito-Keima. After two days transfection, cells were subjected to FACS analysis, counting 10,000 cells per experiment. (A) represents the shift of the signal intensity in USP8 down-regulating cells when excited at 560 nm. (B) Representative scatterplots depicting the mean relative level of global mt-Keima signal in Control (left panel) and USP8 down-regulating cells (right panel).

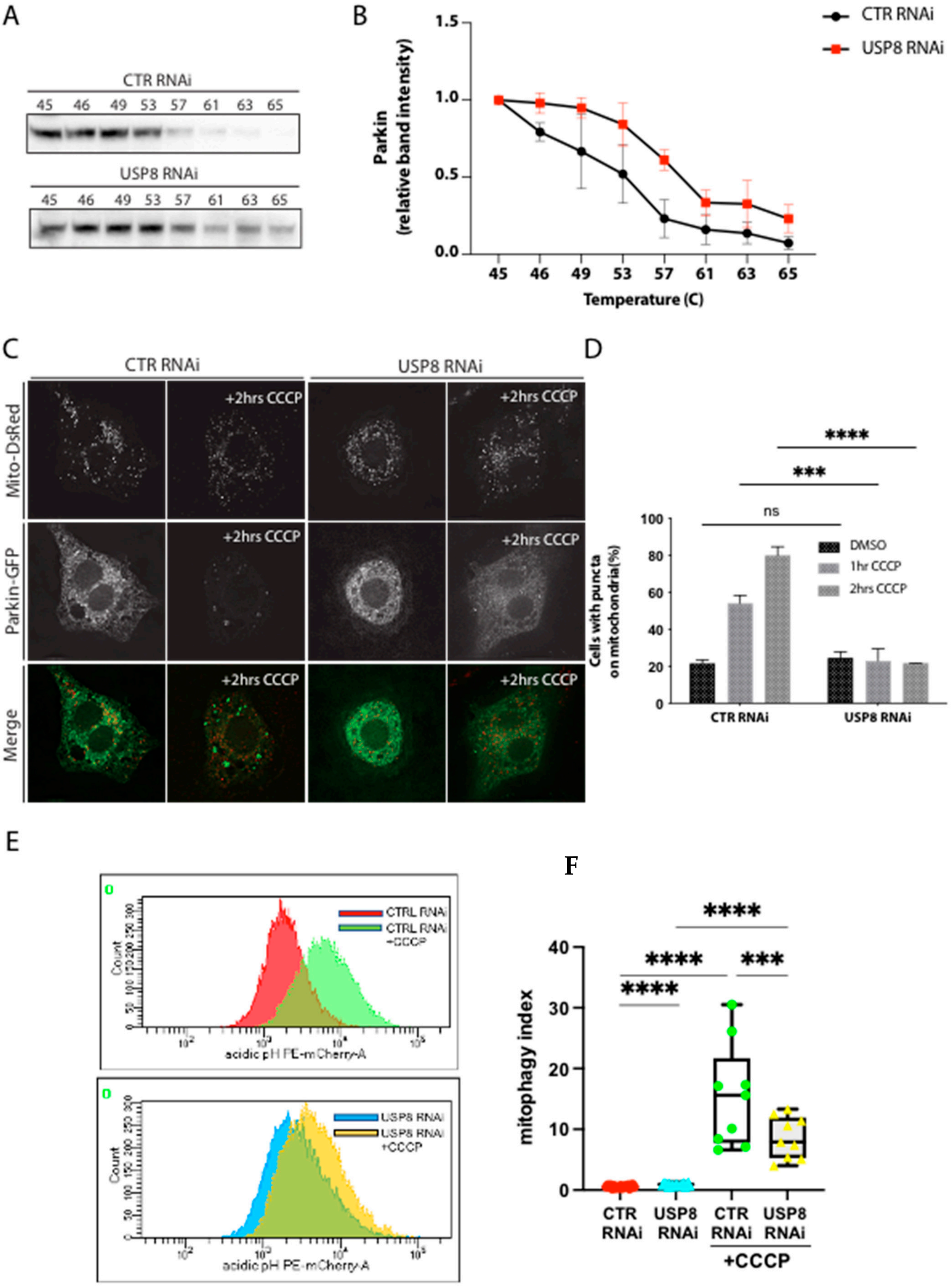
A
CTR RNAi
45 46 49 53 57 61 63 65
USP8 RNAi
45 46 49 53 57 61 63 65
B
CTR RNAi
USP8 RNAi
Parkin (relative band intensity)
1.5
1.0
0.5
0.0
45 46 49 53 57 61 63 65
Temperature (C)
C
CTR RNAi
USP8 RNAi
Mito-DsRed
Parkin-GFP
Merge
+2hrs CCCP
D
****
***
ns
Cells with puncta on mitochondria(%)
100
80
60
40
20
0
DMSO
1hr CCCP
2hrs CCCP
CTR RNAi
USP8 RNAi
E
CTRL RNAi
CTRL RNAi +CCCP
Count
acidic pH PE-mCherry-A
USP8 RNAi
USP8 RNAi +CCCP
F
****
****
***
****
mitophagy index
40
30
20
10
0
CTR RNAi
USP8 RNAi
CTR RNAi
USP8 RNAi
+CCCP

Supplementary Figure S3. (A) Parkin thermal stability assay. Control (CTR) or USP8 downregulating S2R+ cells were suspended in PBS and snap-freezed in liquid nitrogen before being aliquoted into a PCR strip and incubated at the indicated temperature for 3 min. The lysates were centrifugated at high speed and the soluble fraction was loaded into SDS-PAGE gel. Representative Western blotting analysis for Parkin stability is shown. (B) Densitometric analysis of (F). Chart shows mean ± SEM of n = 3 replicates. Statistical significance was determined by two-way ANOVA, followed by Sidak's multiple comparisons test; *** = P < 0.001, **** = P < 0.0001 (C) Parkin recruitment to mitochondria was assessed by live imaging confocal microcopy in cells expressing Parkin-GFP, in which mitochondria were labeled with fluorescent probe mito-RFP. Data are represented as percentage of cells with Parkin puncta on mitochondria. (D) Quantification of (C). Graph bar shows mean ± SEM of percentage of cells with GFP-Parkin on mitochondria for at least ≥300 cells per biological replicate (n=3). Statistical significance was determined by two-way ANOVA, followed by Sidak's multiple comparisons test; *** = P < 0.001; **** = P < 0.0001; (E-F) Cells were treated with the indicated dsRNA (CTR and USP8), and after 24hrs they were transfected with mito-Keima. After two days transfection, cells were subjected to FACS analysis, counting 10,000 cells per experiment. (E) represents the shift of the signal intensity when excited at 560 nm of Control (upper panel) and USP8 down-regulating cells (lower panel) upon CCCP treatment (10 M/20hrs) to induce mitophagy. (F) represents the average intensity of the mito-Keima protein when excited at 560 nm and the ratio between 560:405 nm. Graphs represent the mean±SEM from at least 9 different sets of experiments.

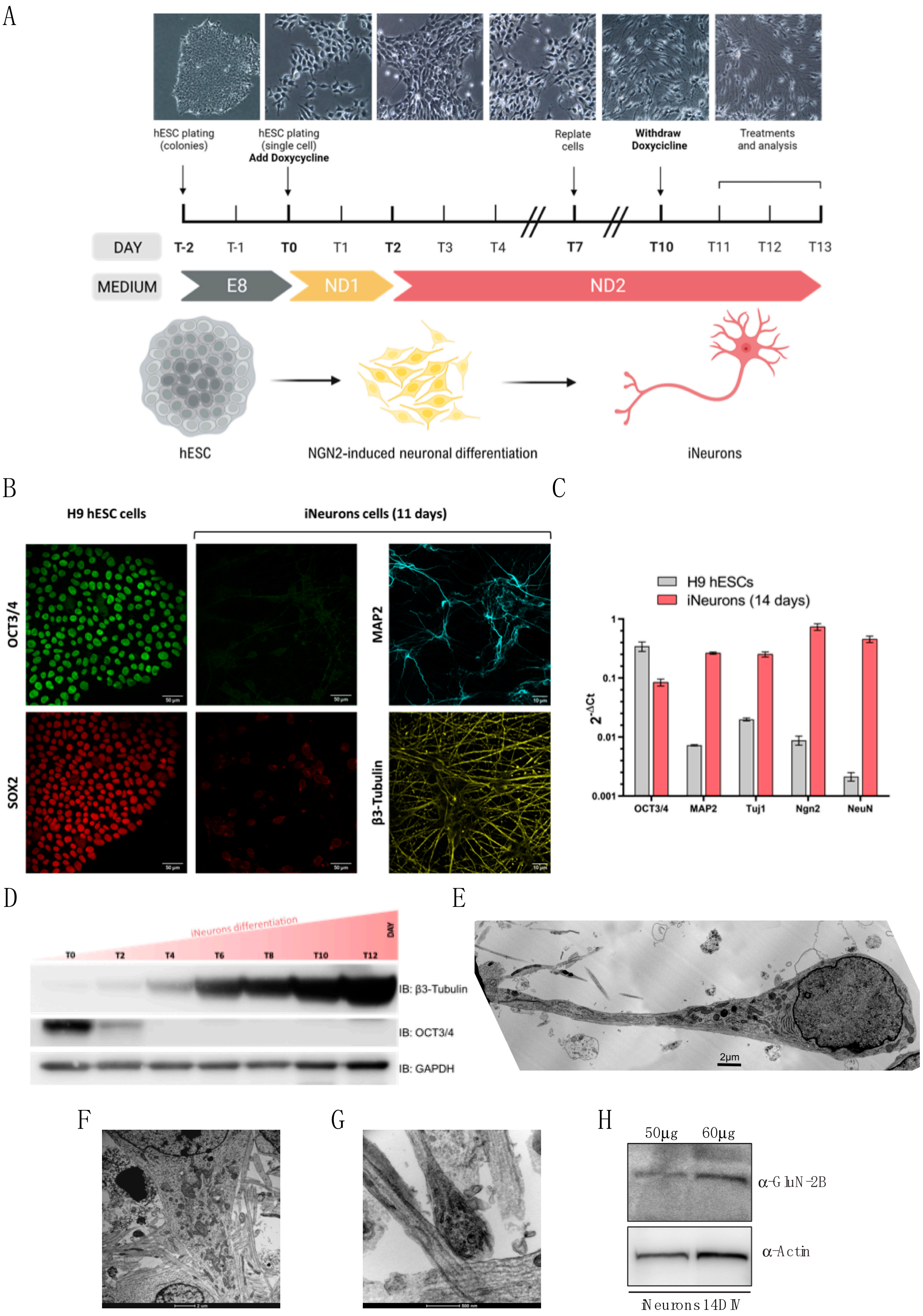
A
hESC plating
(colonies)
hESC plating
(single cell)
Add Doxycycline
Replate
cells
Withdraw
Doxycicline
Treatments
and analysis
DAY
T-2
T-1
T0
T1
T2
T3
T4
T7
T10
T11
T12
T13
MEDIUM
E8
ND1
ND2
hESC
NGN2-induced neuronal differentiation
iNeurons
B
H9 hESC cells
iNeurons cells (11 days)
OCT3/4
SOX2
MAP2
β3-Tubulin
C
H9 hESCs
iNeurons (14 days)
2-ΔCt
1
0.1
0.01
0.001
OCT3/4
MAP2
Tuj1
Ngn2
NeuN
D
iNeurons differentiation
DAY
T0
T2
T4
T6
T8
T10
T12
IB: β3-Tubulin
IB: OCT3/4
IB: GAPDH
E
2µm
F
G
H
50µg
60µg
α-GluN-2B
α-Actin
iNeurons 14DIV

Supplementary Figure S4. (A) Representative images of hESCs undergoing neuronal differentiation. Human neurons are obtained by forcing the expression of transcription factor Ngn2 under the control of a TetO promoter induced by doxycycline. Ngn2 expression in hESCs produces an excitatory layer2/3 cortical neuron that exhibits AMPA-receptor dependent spontaneous synaptic activity and a relatively smaller NMDA-receptor mediated synaptic current. After 4 days of differentiation, cells start to develop a clear neuronal network, and become mature neuronal cells in 14 days. The yield of neuronal conversion is nearly 100%. (B) Representative confocal images of iNeurons stained with the indicated antibodies. At end of the differentiation process (14 days), iNeurons exhibit the expression of the typical neuronal markers MAP2 and βIII-tubulin, and loose pluripotency markers OCT4 and SOX2. (C) Quantitative RT-PCR analyses of the indicated transcription factors. At the end of the differentiating process, iNeurons express ~ 30 to ~ 100-fold increased levels of endogenous Ngn2 as well as of three neuronal markers NeuN, MAP2 and Tuj1. (D) Western blotting analysis of stem cell marker OCT3/4 and neuronal marker βIII-Tubulin. As expected OCT3/4 is only present until day 2 of differentiation while the expression of neuronal marker βIII-Tubulin gradually increased until day 14 upon induction. (E) Representative electron microscopy (EM) image of iNeurons after 14 days of differentiation showing neuronal cells with distinguishable neuronal soma, axon hillock and axonal projection. (F) Enlarged EM image of iNeurons showing detailed axon hillock containing several mitochondrial structures of different size and shape. iNeurons develop dendritic projections (also visible), and mature neuronal network. (G) Enlarged EM image showing released neurotransmitter molecules at synaptic clefts. (H) iNeurons were differentiated from hESCs, and after 14 days of differentiation protein lysates were extracted and protein content (50 µM and 60µM respectively) was subjected to Western Blot to monitor the expression of NMDA receptor(NMDA-R). .
