# Supplementary material for: USP8 Down-Regulation Promotes Parkin-Independent Mitophagy in the Drosophila Brain and in Human Neurons
Source: Cells. 2023 Apr 13;12(8):1143. doi: 10.3390/cells12081143 (PMC10136645; doi:10.3390/cells12081143)
Supplement: Supplementary file 1 [file cells-12-01143-s001.zip › Supplementary Table S1.pdf]

KEGG PATHWAY (USP8 down-regulating flies)

Index	Name	p-value	Adjusted p-value	Z-score	Combined score
1	Homologous recombination	0.002971	0.03743	-74.68	434.55
2	Dorso-ventral axis formation	0.0004092	0.008594	-47.90	373.66
3	Hedgehog signaling pathway	0.01231	0.07754	-61.20	269.11
4	Glycolysis / Gluconeogenesis	0.03123	0.1474	-74.52	258.30
5	Glycerolipid metabolism	0.09940	0.2853	-70.00	161.60
6	AGE-RAGE signaling pathway in diabetic complications	0.05870	0.1947	-56.70	160.75
7	FoxO signaling pathway	0.009388	0.07393	-33.05	154.28
8	Phototransduction	0.000007766	0.0004893	-12.60	148.22
9	Mitophagy	0.07635	0.2405	-50.05	128.75
10	Fanconi anemia pathway	0.03979	0.1474	-30.98	99.90

KEGG PATHWAY (WT flies)

Index	Name	p-value	Adjusted p-value	Z-score	Combined score
1	Amino sugar and nucleotide sugar metabolism	0.008746616	0.107874936	-65.87169767	312.1717947
2	Glycerophospholipid metabolism	0.023585168	0.120622009	-75.27081342	282.0500673
3	Dorso-ventral axis formation	0.012207731	0.109553536	-45.03592726	198.4141482
4	Homologous recombination	0.059470646	0.200037626	-61.58662465	173.8142335
5	DNA replication	0.022325337	0.120622009	-42.17468665	160.3495521
6	Basal transcription factors	0.029645301	0.121875124	-43.60530696	153.4231648
7	Longevity regulating pathway	0.069736187	0.206419112	-56.96589503	151.7022247
8	Fatty acid biosynthesis	0.012251697	0.109553536	-29.1351946	128.2557727
9	Phototransduction	4.44125E-05	0.003286528	-12.59783696	126.2553775
10	Selenocompound metabolism	0.014804532	0.109553536	-29.52368736	124.3780378
